# Prevailing familial, social and cultural obstacles in keeping tobacco-free homes in urban areas of Bangladesh: A mixed-method study

**DOI:** 10.1371/journal.pone.0220777

**Published:** 2019-08-12

**Authors:** Md. Imdadul Haque, ABM Alauddin Chowdhury, Muhammad Shaikh Hassan, Hafiz T. A. Khan, Md. Golam Dostogir Harun

**Affiliations:** 1 Department of Public Health, Faculty of Allied Health Sciences, Daffodil International University, Dhanmondi, Dhaka, Bangladesh; 2 Center of Excellence for Health Systems and Universal Health Coverage, BRAC James P Grant School of Public Health, BRAC University, Dhaka, Bangladesh; 3 The Graduate School, University of West London, London, United Kingdom; Bielefeld University, GERMANY

## Abstract

**Background:**

Millions of children and others across the world are being dangerously exposed to tobacco smoke and toxins in their own homes. Whilst there is limited interest in laws and interventions controlling tobacco use in public places in Bangladesh, no attention has been given to preventing tobacco-use inside homes. This study explores the familial and socio-cultural factors that provide obstacles for ensuring tobacco-free homes in Bangladesh.

**Materials and methods:**

A mixed-method design was adopted and from among the 1,436 tobacco users identified in a population of 11,853, 400 (tobacco users) were selected for cross-sectional survey. This survey involved a probability proportional sampling procedure, and 24 In-Depth Interviews. Multivariate logistic regression was performed to explore the association of familial and socio-cultural factors with tobacco-use at home adjusted by other demographic characteristics. Thematic content analysis was done on the qualitative data, and then inferences were drawn out collectively.

**Results:**

This study revealed that the prevalence of tobacco-use in the home was 25.7% in urban residential areas in Bangladesh. Multivariate logistic regression analysis identified that familial and socio-cultural factors were significantly associated with tobacco-use at home: marital status (OR 3.23, 95% CI: 1.37–6.61), education (OR 2.14, 95% CI: 1.15–3.99), smoking habits of older family members (OR 1.81 95% CI: 0.91–2.89), tobacco being offered as hospitality and for entertainment (OR 1.85, 95% CI: .94–2.95) and lack of religiosity practice (OR 2.39, 95% CI: 1.27–4.54). Qualitative findings indicated that social customs, lack of religious practice, tobacco-use of older family members, and lack of family guidance were key obstacles for enabling tobacco-free homes in urban areas.

**Conclusion:**

Use of tobacco at home is continuing as part of established familial and socio-cultural traditions. If tobacco-use at home is not addressed seriously by the authorities then the emerging threat of second-hand smoke exposure and harmful consequences of tobacco- use will be exacerbated.

## Background

More than 5 million deaths worldwide can be attributed to direct tobacco use, while more than 600,000 are the result of non-smokers, especially women and children, being exposed to second-hand smoke (SHS) [1] usually at home. Throughout the history, a substantial relationship has been identified between social and cultural traditions and tobacco use in many parts of the world especially in Asian and African countries, where tobacco is culturally acceptable [2]. Among Native Americans, for example, tobacco is used for spiritual purposes and even for healing [3]. In the South American Indian tradition, tobacco, is used for purification, connection with the divine, and recreation, and is considered as the social drug in many of their cultures [4]. However, cultural attitudes to tobacco use differ in terms of gender, religion, ethnicity, and local beliefs within the countries [2], and can often act as social obstacles in keeping homes tobacco free. Tobacco use at homes is very high in South Asian countries. Bangladesh is one of the largest tobacco producing and consuming countries in the world [5] and as a result, faces considerable health and economic consequences [5]. Smoking in health care facilitates and educational institutions in Bangladesh, are prohibited by law, and there is a partial ban in public places. However, 43.3% of Bangladeshi adults use tobacco in smoking and/or in smokeless forms. More than 40.0% of young people (age 13–15) are exposed to second–hand smoking (SHS) in public places, and 63.0% of workers overall are exposed to SHS at their indoor work place outside of their homes. There is no national data available on SHS exposure among people in their own homes. [6].

The World Health Organization’s Framework Convention on Tobacco Control (FCTC) concluded that 100% smoke-free environments are the only proven way to adequately protect the health of people from the harmful effects of second hand tobacco smoke because no level of exposure is acceptable [7]. An emerging issue at this time is the so-called 'third-hand smoke' (THS) [8]. THS creates risks for nonsmokers, especially children, who spend time indoors in proximity to polluted surfaces [9]. It is a matter of great concern even where a home’s ventilation system may be strong. Smoke-free laws have been positively associated with people quitting smoking and in preventing young people from initiating to smoke [1]. However, existing tobacco control policies in Bangladesh are still not making any great inroads at the household level.

Smoking prevalence is highest in urban areas in Bangladesh and identified as a rising trend with increased urbanization [10]. Studies have shown that urban dwellers are more aware than rural people about the health consequences of tobacco use, however often they don’t take it seriously and use tobacco at home [10–11]. Many social customs and perceptions influence the behavior of young smokers. For example, when gathering together, they will overestimate the extent of smoking in their own age group so giving them a distorted sense of what is normal behavior [12]. Despite scientific evidence about the harmful effects of smokeless tobacco (SLT) [13], people do not generally believe that the commonly used SLTs such as *Zarda*, *Gul*, *Sada Pata*, (Tobacco leaves as powder form usually chewed with betel leaf in processed form) and many other forms of smokeless products are actually tobacco. Also, the use of SLT in a family and at the household level is a Bangladeshi cultural tradition that is widely accepted and will be served to guests as part of cultural celebrations [13]. Such social customs and cultural traditions act as social obstacles over generations and prevent homes from being tobacco free environments. Social customs and traditions of tobacco use are particularly prevalent in homes in urban residential areas but this is often overlooked [14]. Previous studies conducted in Bangladeshi and Indian context have shown tobacco use to be merely part of cultural traditions, but hardly any research has been conducted into how these traditions work as obstacles for establishing tobacco-free homes [10, 12–13, 15–18]. The key objective of this study therefore was to explore the prevailing familial and socio-cultural aspects of tobacco use in urban residential areas of Bangladesh and identify how these aspects act as obstacles for establishing tobacco-free homes.

## Materials & methods

### Study design and setting

The study used a mixed-method approach with quantitative data collected through a cross-sectional survey and qualitative data collected through In-Depth Interviews (IDIs). This approach provided information for comparing, triangulating, and observing real scenarios about obstacles that can prevent the maintenance of tobacco-free homes. Both sets of data were collected from March to October 2016 from four urban residential areas of the Dhaka City Corporation: Mohammadpur Housing Society (Road: 1–6), Sector-6 (Block–A) of Uttara from North City Corporation, plus Road 27 (Old) of Dhanmondi, and Motijheel colony (A.G.B Colony) from the South City Corporation. These four residential areas were specially selected as study clusters as they represented city dwellers from all corners of the Dhaka city geographically and in having advantage of a city life. Preliminary visits were made to the chosen clusters to gather useful information prior to start of the study.

### Study participants and sampling strategy

All the participants chosen for the survey were tobacco users living in the selected urban residential areas and met pre-defined inclusion criteria as follows: any kind of tobacco users (Smoking or smokeless)–only one from each household, aged 18 years and above, both male and female and physically capable and willing to participate in the survey. Participants were also diverse in terms of age, ethnicity, religion, education and economic status and living in recognizably urban residential areas with access to the advantages of city life. The sample size was calculated using the formula:

$n=\frac{z^{2}pq}{d^{2}}$ Where, n = desired sample size, z = 1.96 (at 95% CI), p = Prevalence of overall current tobacco use (smoking or smokeless) among all adults (aged 18 +) in urban areas = 38.1% [3], q = 1-p, d = precision level (5%). So, n = (1.96)^2^ (0.381× 0.619)/ (0.05)^2^ = 361. A 10% non-response of 361 was anticipated and therefore 400 tobacco users were selected from study area.

**Fig 1:**
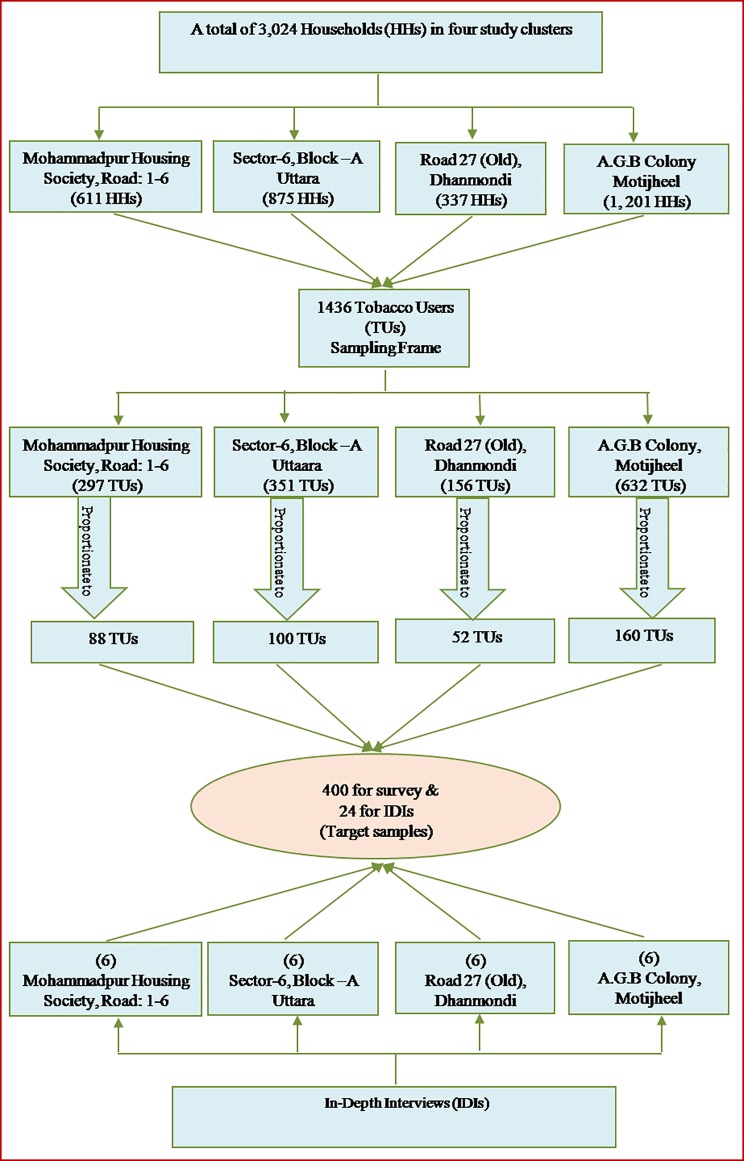
Selecting the target samples (N = 11,853).

Prior to gathering the data, a list of 3,024 households involving a population total of 11,853 was drawn up from the city corporation offices of Dhaka City covering the four study clusters. After a short enumeration survey in these study areas, a list of 1,436 tobacco users was drawn up then used as a sampling frame from which the 400 participants (from 400 households) were identified for data collection. Although four study clusters were specially selected, probability-proportional-to-size (PPS) sampling was used to draw out the target population. The list of tobacco users was separated following 297, 351,156, and 632 respectively for the four study clusters that were then used as four single sampling units of tobacco users, and then from these 400 participants were proportionately gathered. One in every third tobacco users was chosen as a participant. It is noted that around one-third of the people in the sampling frame were not available during the data collection period, so the next participant in the frame who fulfilled the inclusion criteria was selected. (Fig 1). Convenient times were established with participants in ordered to conduct the interviews with at least two revisits necessary in order to reach the required sample number.

In collecting qualitative data, 24 IDIs were conducted to supplement the findings of the survey. The IDIs were helpful for probing into and cross-checking with data generated from the survey, and also for exploring comprehensive and subjective experiences, biographies, beliefs and motivations of tobacco users and non-users for highlighting possible obstacles in creating tobacco-free residential areas.

A snowball technique was used to identify the 24 IDI participants (6 from each cluster) that helped reach those that were considered difficult to access [19]. As mentioned, this study selected influential urban participants and was carried out with a diverse range of both tobacco users and non-users in terms of age, ethnicity, religion, education and economic status. This approach was used in order to achieve a balanced group of participants such as local elites, the higher educated, knowledgeable, and representatives of organizing committees such as in housing plus *Imams* (religious leaders), clinicians, school teachers, businesspersons etc. Along with the Field Supervisor, the Principal Investigator (PI) conducted the in-depth interviews. They were both formally trained in qualitative data collection by Bangladesh Center for Communication Programs (BCCP) that was also the technical supporter of the study. During the preliminary visits to the study areas prior to the main data collection activity, a rapport was established with local community leaders; some of who were eligible to be study participants and data from them was collected first. Local leaders were also able to help identify other participants in the locality. It was discovered that tobacco users among urban residents were often reluctant to admit to their tobacco habits.

### Development of tools, data collection, coding and analysis

As this study involved both qualitative and quantitative methods, a multidisciplinary team including social scientists, epidemiologists, public health specialists, and statisticians contributed to the development of the questionnaire and the in-depth interviews guidelines. The PI and Co-Investigator both had a pivotal role in drafting the questionnaire that was then checked by the other team members and finalized by the technical expert team (BCCP) of this study. A semi-structured interview questionnaire was developed to gather quantitative data. The Bangla version of the survey questionnaire was pre-tested among 20 eligible people (a quarter were female) and qualitative guidelines pre-tested among 4 eligible people (one female) in a non-sample site in an urban residential area within Dhaka City. This enabled feedback to be gathered on the suitability, appropriateness and sequencing of the questions. The survey data were checked for errors, then coded and entered into a database using SPSS software. Analysis of the data was based on the study objectives and statistical tests like Chi Square (*χ*^*2*^) test (Fisher’s exact test used while expected cell value <5), and bivariate logistic regression was used to explore the factors surrounding tobacco use at home. A multivariate logistic regression was performed in order to adjust the effect of confounders on the association of risk factors; a response of “Yes or No” to the question of ‘tobacco use at home’ was a dependent variable, where “No” was used as reference. Socio-cultural and familial factors were used as independent variables, and the findings were interpreted using odds ratio with 95% Confidence Interval (CI) for each category.

The IDIs guidelines and specific data collection techniques were thoroughly reviewed prior to commencement of qualitative data collection to ensure the quality of the data. The IDI guidelines were based on questions used in the quantitative questionnaire to enable exploration of the findings that emerged from the questionnaire. Contemporary literatures on social and cultural obstacles and the consequences for creating tobacco-free urban homes were also reviewed. The average duration of the in-depth interviews was 45 to 90 minutes and usually began with a discussion about the individual, familial and social factors associated with tobacco use. Perceptions around governmental laws and policies related to tobacco intake, roles and initiatives of GO/NGOs to prevent tobacco use at the both individual and community level were also explored. This guideline supported this activity by enabling the interviewer to be flexible to vary and probe responses more deeply as interesting aspects of tobacco use emerged. The guidelines were also followed in preparing the final study report.

Qualitative data was coded separately. Raw data were gathered during the IDIs on tape recorders that were then transcribed in standard Bangla and information from notes written during the interviews was also included. The research team edited the raw data and rearranged it, manually coded it, and then themes and relevant quotations were identified on a daily basis. After coding, the data were translated into English and thematic content analysis was performed. Similar concepts that emerged from thematic analysis were identified and drawn together to from common themes and sub-themes. [19]. In this way, although the IDIs were analyzed separately, inferences were drawn collectively from the results. Key findings are presented in this paper involving the social, familial and cultural obstacles that can prevent homes from being tobacco-free over time.

### Ethical considerations

The study protocol was reviewed and approved by the National Research Ethics Committee (NREC) of the Bangladesh Medical Research Council (BMRC). Prior to starting the data collection, the interviewers briefed participants about the background and objectives of the study and each participant signed an informed consent form before an interview commenced. Anonymity and confidentiality were strictly maintained.

## Results from quantitative analysis

### Socio-demographic characteristics of the participants

The mean age (± SD) of participants was 30.4 ± 10.4 years. Higher age group (30 years and over) among the participants tended to use more tobacco products at home than the youngsters. Age was found to be significantly associated (P<0.001) with place of tobacco use.

Majority (84.6%) of the female tobacco users used tobacco products at home in the study areas. Furthermore, higher proportion of females are engaged in tobacco use at home compared to males. There was a highly significant association (P<0.001) between sex and place of tobacco use. Similarly, marital status had a highly significant association with place of tobacco use (P<0.001), where more married participants (25.4%) found to use tobacco products at home compared to their unmarried counterparts (9.1%). Higher proportion (27.1%) of participants coming from a joint family were using tobacco at home compared to participants from a nuclear family (14.3%), and this association was statistically significant (P<0.01). Lower and middle educated participants were more likely to report tobacco-use at home than the educated group and the association was statistically significant with P<0.001 (Shown in Table 1).

**Table 1:** Socio-demographic characteristics of the participants by their place of tobacco consumptions.

Demographic characteristics	Place of Tobacco use by participants = 400		*χ*^2^*	P value
	At home n (%)	Outside home n (%)		
**Age**				
≤ 30 Years	28 (10.7)	234 (89.3)	25.94	<0.001
>30 Years	43 (31.2)	95 (68.8)		
*Mean ± SD* *30*.*4 ± 10*.*4*				
**Sex**				
Male	60 (15.5)	327 (84.5)	41.14	<0.001
Female	11 (84.6)	2 (15.4)		
**Marital status**				
Unmarried	17 (9.1)	170 (90.9)	18.03	<0.001
Married	54 (25.4)	159 (74.6)		
**Living place**				
With family	54 (18.2)	243 (81.8)	0.15	0.112
Alone/Outside family	17 (16.5)	86 (8.5)		
**Family type**				
Nuclear Family	42 (14.3)	251 (85.7)	8.75	0.002
Joint Family	29 (27.1)	78 (72.9)		
**Education**				
Primary- Secondary	29 (23.8)	93 (76.2)	11.86	<0.001
Higher education	42 (15.1)	236 (84.9)		
**Socio-economic condition**				
Low and middle income	21 (19.3)	88 (80.7)	0.24	0.102
Upper and high income	50 (17.2)	241 (82.8)		

* Fisher’s exact test was used as some of the expected cell value (for sex) found <5.

### Prevalence of tobacco use at home (household level)

**Fig 2:**
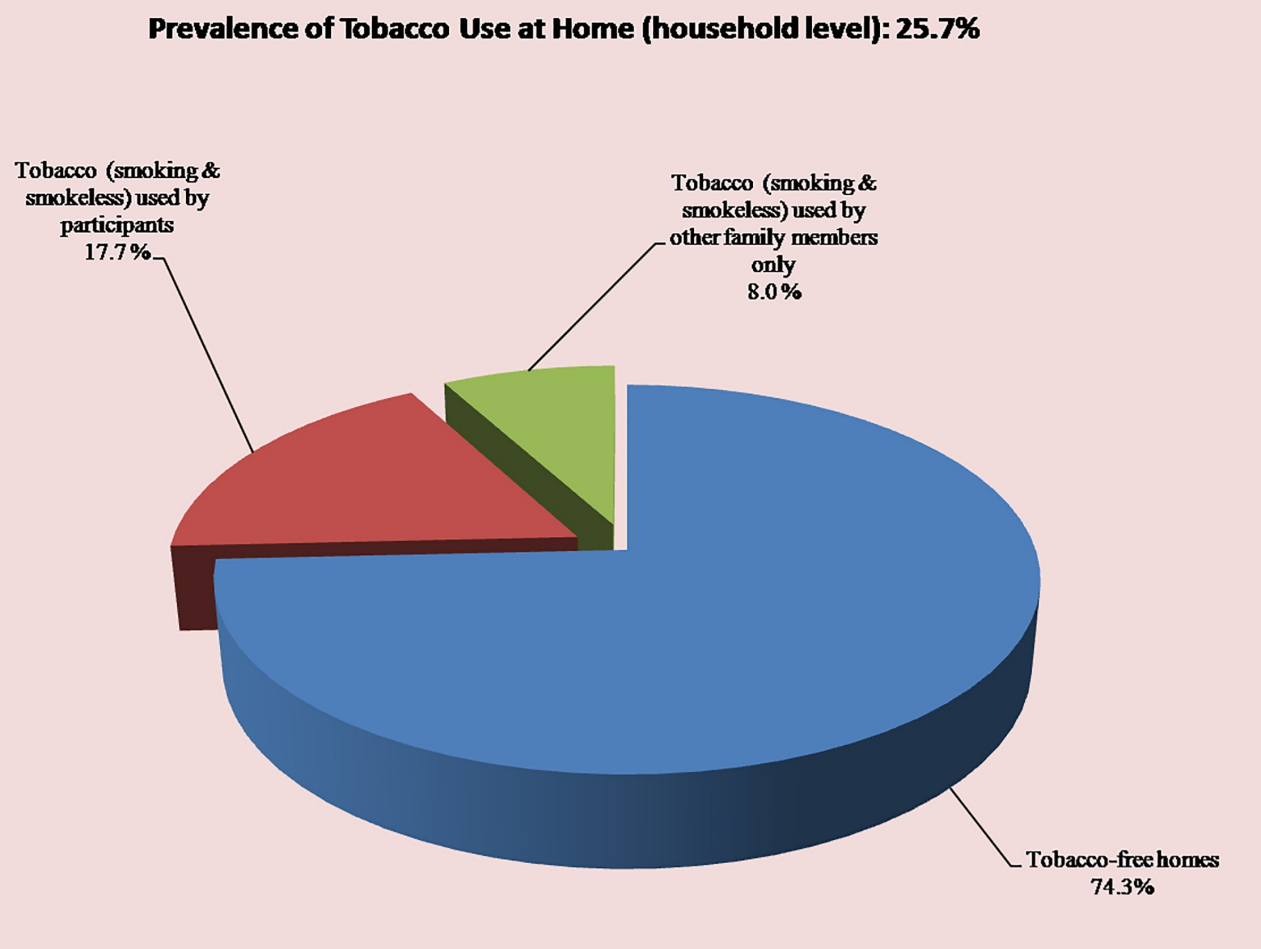
Prevalence of tobacco use at home by participants and other family members in the study areas (At household level) (n = 400).

The primary unit of interest in this study was the household. The prevalence of tobacco use at home was calculated by dividing the total number of persons (either participant or any other family member), who used tobacco products inside homes with all sample households. The study procedure considered only one tobacco user from each household. Thus, home was chosen by 17.7% of the participants as their usual place for consuming tobacco products (smoking or SLT). Around 8.0% of other family members of participants used some kind of tobacco (smoking or SLT) at home. Overall, the prevalence of tobacco use at the home was calculated to be 25.7% in urban Bangladesh (Fig 2). This meant that is, more than one-fourth of households had at least one tobacco user, who usually chose home to pursue their habit.

### Risk factors for tobacco use at home

The study aimed to explore the risk factors of tobacco use at home. From bivariate analysis, it was observed that age, marital status, education, religiosity practice, smoking habit of any older family members, children used to carry/buy tobacco and tobacco offering as a means of entertainment at home were all associated with using tobacco products at home (Table 2). In the multivariable analysis, after adjusting for possible confounders, some factors were found to be significantly higher in contributing towards tobacco use at home. Participants aged >30 years (OR = 3.13, 95% CI 1.45–6.78) were more than three times likely to use tobacco products at home compared to those aged ≤30 years.

**Table 2:** Adjusted risk factors associated with place of tobacco use in urban Bangladesh.

Characteristics/ Risk factors		Bivariate analysis OR (95% CI)	P-value	Multivariate analysis OR (95% CI)	P-value
**Age**	≤ 30 Years	1		1	
	>30 Years	3.78 (2.22–6.44)	<0.001	3.13 (1.45–6.78)	0.004
**Marital Status**	Unmarried	1		1	
	Married	3.39 (1.89–6.10)	<0.001	3.23 (1.37–6.61)	<0.001
**Socio- economic condition**	Low and middle income	1		1	
	Upper and high income	1.15 (.65–2.02)	0.627	.66 (.33-.1.30)	0.234
**Living status**	Living with family	1		1	
	Living alone/others	1.12 (.62–2.04)	0.701	. 69 (.35–1.37)	0.298
**Education**	Higher education	1		1	
	Primary- Secondary	2.46 (1.46–4.16)	<0.001	2.14 (1.15–3.99)	0.016
**Family type**	Nuclear family	1		1	
	Joint family	.45 (.26-.77)	0.004	.49 (.28-.85)	0.0490
**Occupation**	Non-working	1		1	
	Working	.40 (.21-.75)	0.005	.96 (.44–2.12)	0.932
**Practice of Religiosity**	Practice	1		1	
	Lack of Practice	2.25 (1.20-.4.21)	0.011	2.39 (1.27-.4.54)	0.019
**Smoking habit of any elder family members**	No	1		1	
	Yes	1.97 (1.28–2.28)	<0.001	1.81 (0.91–2.89)	0.031
**Smoking at home as Looks smart**	No	1		1	
	Yes	.79(.47–1.35)	0.402	.61 (.34–1.07)	0.089
**Tobacco restriction at home**	No	1		1	
	Yes	.66 (.40–1.13)	0.140	.70 (.40–1.21)	0.202
**Children are used to buy/carry tobacco/lighting cigarette**	No	1		1	
	Yes	2.07 (1.14–3.79)	0.017	2.28 (1.21–4.29)	0.148
**Lack of family guidance**	No	1		1	
	Yes	.89 (.36–2.21)	0.798	.94 (.35–2.46)	0.903
**Tobacco offering as a means of entertainment at home**	No	1		1	
	Yes	1.81 (.94–3.51)	0.003	1.85 (.94–2.95)	0.004
**Peer influences (smoking)**	No	1		1	
	Yes	.49 (.14–1.67)	0.257	.41 (.11–1.45)	0.406
**Impact of advertisement and publicity**	No	1		1	
	Yes	1.29 (.77–2.16)	0.337	1.31 (.76–2.26)	0.325

OR* = Odds Ratio; Reference category = Tobacco use at home

The odds of tobacco use at home among married participants (OR 3.23, 95% CI: 1.37–6.61) were also found to be more than three times than their unmarried counterparts. Use of tobacco at home was likely to double in the households where there was the custom of offering tobacco to the guests and visitors compared to those didn’t offer. (OR 1.85, 95% CI: .94–2.95). Lower educational status (OR 2.14, 95% CI: 1.15–3.99), and lack of religiosity practices among participants (OR 2.39, 95% CI: 1.27–4.54) recorded more than two times proximal with using tobacco products at home. The risk of tobacco use at home became a little higher among those family members, where there was a smoking habit in an older family member (OR 1.81 95% CI: 0.91–2.89). However, the multivariate analysis showed that many other factors such as peer influence (for smoking), occupation, living status, family type, looking smart, tobacco-use restricted at family level were found to be insignificant factors for using tobacco products at home (Table 2).

## Results from qualitative analysis

### Thematic content analysis of IDIs

Data from the 24 in-depth interviews were arranged into themes. Participants were asked about obstacles that they considered prevented them from keeping their urban residential homes tobacco-free. Nine key themes were identified that were then categorized into four areas: three social factors, one cultural-religious factor, three familial factors, and one combined factor that included:: 1) peer influence; 2) social myth and customs regarding tobacco use; 3) negligence of knowledgeable people on the harmful consequences of smoking; 4) limited roles of mass media; 5) lack of religious practices; 6) tobacco use of older family members at home; 7) lack of family bonding; 8) Lack of family guidance in childhood and 9) lack of societal pressure.

#### 1. Peer influence

The finding about peer influence on tobacco use was found to be inconsistent with the survey results. Though peer smoking may be initiated outside of the home, due to its addictive nature, it can influence the desire to start smoking at home later on, whereas SLT is culturally acceptable over time at the household level and therefore create social obstacles in establishing a tobacco-free home. Smoking seemed to be regarded as a sophisticated activity to explore personal enhancement among the peers, and not considered within a circle of friends as a cause of any harm.

*“I found*, *people mostly initiate smoking to show them as more fit, modern, smart, and sophisticated in friends circle, and this tendency is more frequent among young population and student group in our country.” (Cluster-2, IDI-4)*

*“I started smoking from my high school life*. *Friend circles*, *who were habituated smoking*, *often provoked me–let’s puff once*, *that will make nothing happen*. *I got addicted to taking one-two puffs with influences of them*, *even though none of my family members smoked”*. *(Cluster-3*, *IDI-2)*

Participants also reported that the influence of peers were even more effective in encouraging them to take up SLT products.

*“…Those who are smokeless tobacco users have psychic unity*, *not necessary to be acquainted with to share tobacco products irrespective of their age and gender” (Cluster-2*, *IDI-1)*

#### 2. Social myths and customs regarding tobacco use

A number of young participants thought that smoking invigorated energy, increased their working capacity and made people that indulged in it look smart. Such social myths and customs were identified for both smoking and SLT use. Smoking is often overestimated in the community, and in the case of SLT consumptions, the established notion is that its use is not harmful rather, it can help reduce bad breath, for instance.

*“I don’t know the fact of others, but in my case, whenever I was stressed with working load in office or home, and I smoke two cigarettes consecutively. I think, it instantly makes me light and easy*, *feels refreshed and new spirits that help continue my works”. (Cluster-3, IDI- 4)*

SLT products were used extensively in the homes of participants to indicate hospitality as a cultural tradition. If a visitor were in the home, he or she would be offered betel leaf with SLT.

*“Hey! Since time immemorial, we entertain a guest with betel leaf with Jarda (smokeless tobacco) and still in our cultural functions and occasional festivals*, *we first offer people betel leaf. It is our tradition; we enjoy and share it always among us… (Smokeless tobacco users)” (Cluster-4, IDI-1)*

#### 3. Negligence of knowledgeable people on the harmful consequences of smoking

Although many participants were educated and socially established, their perceptions about tobacco were not clear, and they had become used to tobacco products. Such people knew about the harmful effects of smoking, but continued to smoke, and therefore, were not in a position to advise others to abstain from doing so. Being educated, they stood as role models for other family members at home, especially younger members, who would then follow their lead.

*“By the way, the leading problem in reducing the smoking is the shortage of awareness. Most of the educated people, more or less, know the harmful physical consequences of smoking; they neglect the harmful consequences, and negatively perceived”*. *(Cluster-3, IDI-4)*

#### 4. Limited roles of mass media

Many people in these times are busy on social media and using electronic devices such as mobile phones, computers, television etc. that has resulted in a significant decrease in person-to-person interactions and social gatherings. Technology cannot effectively share stories of wellbeing or woes, and are not effective in preventing a person from becoming addicted to smoking or other drugs and, indeed, can often work negatively in promoting smoking products.

*“I saw in many dramas and cinema, where message discouraging smoking was publicized, but showed the scene of publicly smoking. Children imitate these scenes later on in their life, negatively act at their personality”*. *(Cluster-4, IDI-1)*

In regard to the role of mass media, participants remarked:

*“They (Electronic media) don’t air any news regarding tobacco intake, and whatsoever aired, by which people are not inspired to give up tobacco products and failed to contribute effectively in quitting tobacco consumptions….”(Cluster-2*, *IDI-3)*

#### 5. Lack of religious practices

Participants commented that it was easier to make a home tobacco-free, but very difficult to do so for a whole community. With this in mind, a number of participants endorsed the notion that beliefs and practices of religiosity could have an impact, as they believed that all religions discourage smoking, or any types of drug addiction.

*“We, first, have to be tobacco-free, make our family members practice religiosity and moralities and provide in-depth ideas about the ultimate outcome of tobacco using. That is how, if we start at the family level*, *I think, we can shape someday our housing areas tobacco-free”. (Cluster-4, IDI-3)*

Other participants thought that those in well-off families living in urban areas were more engaged in cultural practices and extra-curricular activities such as singing and dancing, for example, that they had indulged in from childhood rather than practicing religiosity early in life.

*“I myself experienced earlier life in the village, children are sent to moktob (informal religious education center), where they could learn the lessons of good courtesy and religiosity, which is less likely found in the urban areas*. *However, these lessons in childhood keep auxiliary and directed them with good demeanor later on in their life and help abstain from taking tobaccos or addictions in any type”. (Cluster-1, IDI-2)*

#### 6. Tobacco use of older family members at home

Participants identified the negative role of older family members and so a more positive input from them was seen as crucial for controlling the extent of tobacco use in the home. In most cases, parents, older siblings, grandparents and other older members of the family smoked or used tobacco products in front of children that encouraged initiating tobacco use by those children in the future.

*“…no advice or no efforts of controlling tobacco uses among the family members will work, unless elderly family members abstain from tobacco using” (Cluster-1*, *IDI-4)*

#### 7. Lack of family bonding

Ties among family members were reportedly found to be weaker in the urban well-off families, and there were many instances in such affluent families where the parents frequently smoked, took drugs, went to bars, and settled divorces badly. Children from such families also became addicted to smoking and other drugs from the adolescence.

*“I saw in some families in residential areas, where parents often move to opposite direction in the family, and children are not fostered in controlled atmosphere, where they enjoy extreme freedom, and ultimately with the passage of time, children get controlled by the friends or extraneous surrounding environment that ultimately accompanied by the influence of their fellow friends; they are unwittingly addicted to smoking and some higher drugs.” (Cluster-1*, *IDI-2)*

#### 8. Lack of family guidance in childhood

Family guidance in childhood about the negative effects of tobacco use was found to be very significant in dealing with any obstacles. Unfortunately, very few parents were found to be educating their children from their early years about the harsh consequences of tobacco use.

*“Those who smoke*, *go and investigate them*, *they have no good relationship within the parents and children as well in the family*, *and received no moral education from the family on the bad consequences of tobacco intake”*. *(Cluster-1*, *IDI-6)*

*“…I think… uhh…elder family members themselves not only should keep away to use tobacco products, but also they should give proper lessons regarding the harmful consequences of tobacco uses to their younger members by different family interventions in a different fashion”*. *(Cluster-3, IDI-4)*

#### 9. Lack of societal pressure

A majority of participants recorded that combined efforts from every level of society to stop tobacco use was not drawn up by people living in the communities or by the authorities concerned. It was believed not to be possible to help people stop using tobacco simply by making laws, as they would still not be aware of the consequences of their actions.

*“To me …tobacco free home can be managed, if the entire population of housing society took measures against tobacco uses in the community and jointly say ‘no’ to all the tobaccos products”*. *(Cluster-1, IDI-4)*

*“…the tobacco controlling endeavors never sees its success as it is not initiated in every part of the government from their respective areas, as well. Medias can air various awareness making programs for the people to be conscious, the text books can draw the scenarios of threatening instance of smoking, various short stories, poetry, cinemas*, *or songs can be made with motivational dimension, even Imam in the mosque can discuss in his khutba (weekly prayers time) about the health and economic burden, and about violence of moralities by tobacco using”. (Cluster-3, IDI-2)*

## Discussion

The indoor and outdoor environmental impact of tobacco use is huge with developing countries in particular paying an innumerable cost for continued indoor tobacco use. Existing literature, policies and initiatives pay little attention to tobacco-free homes in Bangladesh even though home is the place that produces large scale SHS smoke exposure that causes harm to children, women and elderly people [6, 20]. Studies on second hand smoking identified that inhaling second-hand smoke is around four times more toxic and side stream condensate is two to six times more carcinogenic than mainstream [21–23]. However, Tobacco (both smoking and SLT) is commonly used after having food, snacks, tea in small and large social gatherings, as a cultural practice of Bangladeshi people that extends back over the centuries, and was shown in a study conducted in urban areas of Bangladesh [15–16] with similar scenarios in many other studies focused on rural areas [13, 17–18]. All these studies delineated tobacco use, as part of Bangladeshi social and cultural practices apparent in every sphere of life over generations and urban areas were not exception.

This study is potentially the first to document the prevalence of tobacco use at home in urban residential areas of Bangladesh. More than one-fourth (25.7%) of tobacco users (either smoking or smokeless) chose home as their usual place for tobacco use at household level and did not give any thought as to the potential harm their action could cause to other family members. These results found in harmony with the findings of a community-based study conducted in rural setting in Bangladesh, which showed that smoking inside the home was common practice with more than half (55.0%) of households having at least one smoker [24]. Similar trend was also evident in the neighboring country India, where 40.0% of Indian adults reported to smoke tobacco products at home; supposedly for similar socio-cultural setting [25].

Our study distinctively identified various familial, social and cultural factors that have proved to be strong obstacles for creating a tobacco-free residential area. From bivariate and multivariable analysis after adjusting for possible confounders, it was observed that the likelihood of tobacco use at home among the married participants (OR 3.23, 95% CI: 1.37–6.61) were more than three times than their unmarried counterparts. A possible reason for this could be that unmarried family members in Bangladesh would often be dependent, and so less likely to be allowed to use tobacco products at household-level [10, 16–17, 26]. This finding is in line with other study findings conducted in rural Bangladesh that showed married people were more likely to use tobacco products at household level than their unmarried counterparts [17].

From the bivariate and multivariate test (adjusted), the study also examined that the lower educational status of the family members (OR 2.14, 95% CI: 1.15–3.99) significantly contributed to their use of tobacco products at home. This happens because majority of the people with lower educational status have very poor knowledge on the specific health risks of tobacco products; they usually use tobacco as the means of traditional hospitality, removing bad odor of mouth (prefer SLT), and escaping from stress and anxiety [10, 15–17, 26–27]. Similar finding was reported in other studies conducted in low and middle-income countries [28–30]. However, the qualitative findings showed that although a good number people in residential areas are educated and socially established, their perception about using smoking products were not clear. They ignored the harmful consequences, and served as a role models for others to carry on smoking.

The bivariate analysis (unadjusted) further revealed that at family level were significantly, high in the households, where children were used to buy/carry tobacco or to light the cigarette OR: 2.07 95% CI: 1.14–3.79). However, after adjusting for confounders, the multivariable analysis revealed that the risk of tobacco use at home was a little higher among those families, where any of the older family members smoked at home (OR 1.81 95% CI: 0.91–2.89). Aligned to this finding, the qualitative data indicated that in most of the cases, where parents, grandparents and other older family members smoked or used tobacco items in front of children, it latently encouraged tobacco use by those children in the future. This finding accords with another study documented in California, USA that showed teenagers notice older people smoking, including their parents and relatives, and would take up smoking in order to be perceived as older [27]. Other studies also recorded similar findings where parents, older family members and peers had significant influence on teenagers for initiating smoking among them [31–36].

This study did not find any association between restriction on tobacco use among family members and its use at home. This finding was contrasted with other studies conducted in the USA that showed tobacco use was more likely when it was not restricted within the family [37–38].

The qualitative findings explored that lack of family guidance in childhood about the negative effect of tobacco use significantly contributed to children trying tobacco products later in life. This finding is consistent with another study conducted in Vietnam identified that family guidance and interactions related to smoking behaviors had a strong influence on a smoker's intention to quit [39].

In contrast with several studies conducted outside Bangladesh, this study revealed that tobacco use at home was not significantly associated with family members that perceived ‘smoking at home, shows smartness’ compared to those who did not perceive this. However, this study documented that the practice of ‘tobacco offering as the means of entertainment at home’(OR 1.85, 95% CI: .94–2.95) is almost two times risker for use of tobacco products at household level. Offering tobacco to guests and intimate friends who come to visit a home is a traditional practice in Bangladesh, and helps to continue the use of tobacco (especially SLT) products in that environment. The IDIs findings of this study also showed that the social myths and cultural traditions related to tobacco use become embedded over a long period of time and represent a big challenge for effective tobacco controlling interventions in urban areas. Another qualitative study in urban slum areas reported that smokeless tobacco use is the traditional sign of hospitality in Bangladesh and is practiced in various social activities such as marriage ceremonies, cultural, and religious events other occational festivals [26].

Studies conducted outside of Bangladesh explored various social and familial issues associated with tobacco use at home. However, review of past literature reveals that a good number of studies carried out in Bangladesh have already addressed the variety of problems focusing tobacco issues among the Bangladeshi people, but none of them could address the obstacles for ending tobacco use at household level in urban residential areas.

This study indicated that peer influence among family members at the household level was not associated with the risk of tobacco use at home. IDI participants however, indicated that peer influence or peer tobacco use provided serious obstacles for preventing smokless tobacco use, since sharing smokeless tobacco at household level was considered as the means entertainment and hospitality and was a long standing cultural tradition in Banglaesh. IDI participants reported that peer influence outside the home often strongly influenced adolescents to take up smoking that pushed them to use tobacco products at home later on in their lives and would then lead to adiction. Aligned to the findings from the IDIs, many studies have identified that adolescents are greatly influenced by the tobacco use of their siblings, cousins, peers and friends [40–43].

This study finds a significant association of tobacco use and regular religious practice. That is, the participants who were in religious practices regularly (such as praying, giving things to charity, reading religious books etc.) were less likely used to use tobacco at home. Sociologists like Durkheim have long earlier suggested that the role of religion is to exercise control over people's behavior. Individuals with higher levels of religiosity support more restrictive tobacco/alcohol polices; simply because, tobacco/alcohol use is discouraged in almost all conventional religions for its additive nature and explicit physical harms [44–46]. A study conducted in the Dominican Republic identified the similar relationship between smoking and religiosity [47]. In contrast, another study conducted among households in the USA [48] concluded that religion and religious beliefs do not feature prominently for instigating smoking bans in people’s homes. However, in many parts of the USA, tobacco use does not only have a religious factor, rather it is considered to be important in local rituals, and an essential part of the cultural tradition. [3–4].

This study can greatly help to create a primary concern for pursuing a larger study with a broader context covering similar issue. It also demonstrated the need to set out policies for initiating new interventions to reduce the extent of tobacco use at urban household and community level of residential areas in Bangladesh.

### Strengths and limitations of the study

A limitation was the low number of participants (n = 400) across the four urban areas. This was compensated for by information obtained from 24 IDIs. It cannot be claimed that the information based on one city is representative for all urban areas of Bangladesh as a whole in terms of finding obstacles for keeping homes tobacco-free. In addition, due to a very high rate of migration/relocation among Dhaka city dwellers (more than one-third), the study could not enroll some sample HHs during the data collection and had to consider next HHs from the sampling frame. However, by using a mixed-method approach, the findings provided a comprehensive description of the prevailing constraints and barriers that hinder the maintenance of tobacco-free homes in urban residential areas. Generalization of similar scenarios of socio-familial obstacles in creating tobacco-free homes could be applied to other urban areas in Bangladesh.

## Conclusions

It is potentially very alarming that that one-fourth of the urban dwellers use tobacco products at home. Familial factors such as the smoking habits of family members and no restrictions on tobacco use in the family, plus social customs and traditions such as offering tobacco as the cultural means of hospitality/entertainment over generations, and lack of religiosity practice in current-age people have been at work that all helped to continue tobacco use at home. It is, therefore, the high time to enact appropriate laws that would consider homes to be tobacco-free, as experienced in many other countries in the world. If tobacco use at home is not dealt with appropriately, then the dire consequences of second hand smoke exposure will start to emerge in the near future.

## Recommendations

■ As a part of community intervention, anti-tobacco campaign, posters, stickers, or games may be useful to create awareness among the mass people about tobacco use at home in residential areas.
■ Local authorities and GO/NGOs need to come forward with simple family-based interventions/campaigns considering family guidance on the consequences of tobacco use as well as creating a norm of tobacco-free household through strong family bonding, and sufficient recreational system.
■ Regular practice of religiosity and religious initiatives taken by the *Imam* (religious leader in Islam) and clergymen could help to limit the use of tobacco at home.

## Abbreviations

BMRC: Bangladesh Medical Research Council
FCTC: Framework Convention on Tobacco Control
HSC: Higher Secondary Certificate
IDI: In-Depth Interview
PI: Principal Investigator
PPS: Probability Proportion to Size
SD: Standard Deviation
SHS: Second Hand Smoke
SLT: Smokeless Tobacco
SPSS: Statistical Package for Social Sciences
THS: Third Hand Smoke
WHO: World Health Organization
